# *N*-(3-Methyl­phen­yl)benzamide

**DOI:** 10.1107/S1600536808035186

**Published:** 2008-10-31

**Authors:** B. Thimme Gowda, Sabine Foro, B. P. Sowmya, Hartmut Fuess

**Affiliations:** a Department of Chemistry, Mangalore University, Mangalagangotri 574 199, Mangalore, India; b Institute of Materials Science, Darmstadt University of Technology, Petersenstrasse 23, D-64287 Darmstadt, Germany

## Abstract

The asymmetric unit of the title compound, C_14_H_13_NO, contains four mol­ecules, which are linked through N—H⋯O hydrogen bonds into two symmetry-independent chains running parallel to [001] and [101]. The N—H and C=O bonds of the amide groups are *trans* oriented in all four mol­ecules. The mol­ecules are not planar and both aromatic rings are twisted strongly relative to the plane of the amide group. The dihedral angle between the two benzene rings ranges from 70.6 (2) to 74.2 (2)°. The N—H bond is *anti* to the *meta*-methyl substituent in the aniline fragment in three of the four symmetry-independent mol­ecules. In the fourth mol­ecule, the aniline unit is disordered over two nearly coplanar positions; the *anti* and *syn* conformers occupy the same site in the crystal with equal probability.

## Related literature

For the general procedure for the synthesis of the title compound, see: Gowda *et al.* (2003 ▶). For structure of the 3-chloro­phenyl analogue, see: Gowda *et al.* (2008 ▶).
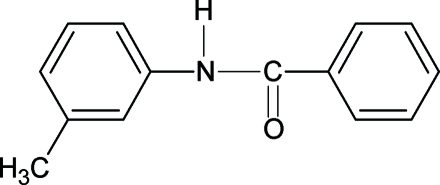


## Experimental

### 

#### Crystal data

C_14_H_13_NO
*M* *_r_* = 211.25
Monoclinic, 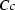

*a* = 13.269 (2) Å
*b* = 53.686 (6) Å
*c* = 9.3921 (12) Å
β = 134.21 (1)°
*V* = 4795.7 (10) Å^3^
*Z* = 16
Cu *K*α radiation
μ = 0.58 mm^−1^
*T* = 299 (2) K
0.43 × 0.25 × 0.10 mm

#### Data collection

Enraf–Nonius CAD-4 diffractometer
Absorption correction: none
9315 measured reflections
4168 independent reflections
3197 reflections with *I* > 2σ(*I*)
*R*_int_ = 0.028
3 standard reflections frequency: 120 min intensity decay: 1.0%

#### Refinement

*R*[*F* ^2^ > 2σ(*F* ^2^)] = 0.043
*wR*(*F* ^2^) = 0.129
*S* = 1.03
4168 reflections
653 parameters
110 restraints
H atoms treated by a mixture of independent and constrained refinement
Δρ_max_ = 0.27 e Å^−3^
Δρ_min_ = −0.13 e Å^−3^

### 

Data collection: *CAD-4-PC* (Enraf–Nonius, 1996 ▶); cell refinement: *CAD-4-PC*; data reduction: *REDU4* (Stoe & Cie, 1987 ▶); program(s) used to solve structure: *SHELXS97* (Sheldrick, 2008 ▶); program(s) used to refine structure: *SHELXL97* (Sheldrick, 2008 ▶); molecular graphics: *PLATON* (Spek, 2003 ▶) and *ORTEP-3 for Windows* (Farrugia, 1997 ▶); software used to prepare material for publication: *SHELXL97*.

## Supplementary Material

Crystal structure: contains datablocks I, global. DOI: 10.1107/S1600536808035186/gk2174sup1.cif

Structure factors: contains datablocks I. DOI: 10.1107/S1600536808035186/gk2174Isup2.hkl

Additional supplementary materials:  crystallographic information; 3D view; checkCIF report

## Figures and Tables

**Table 1:** Hydrogen-bond geometry (Å, °)

*D*—H⋯*A*	*D*—H	H⋯*A*	*D*⋯*A*	*D*—H⋯*A*
N1—H1*N*⋯O2^i^	0.87 (3)	1.96 (3)	2.822 (4)	168 (4)
N2—H2*N*⋯O1	0.84 (3)	2.06 (3)	2.848 (4)	156 (5)
N3—H3*N*⋯O4^ii^	0.86 (3)	2.00 (3)	2.829 (4)	163 (4)
N4—H4*N*⋯O3^i^	0.82 (3)	2.04 (3)	2.813 (4)	156 (5)

Symmetry codes: (i) 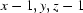; (ii) 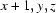.

**Fig. 1.**
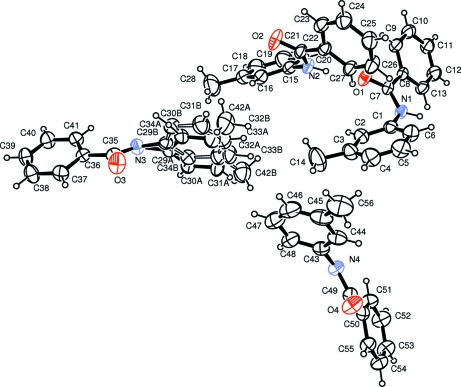
Molecular structure of the title compound showing the atom labelling scheme. The displacement ellipsoids are drawn at the 30% probability level. Both disordered components are shown (the bonds of the second component are drawn with dashed lines).

**Fig. 2.**
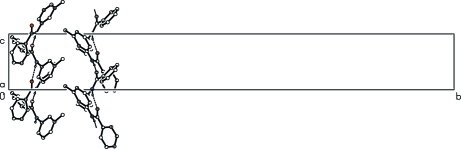
The molecular packing of the title compound, with hydrogen bonds shown as dashed lines. For clarity, the disordered components and the H atoms not involved in hydrogen bonding have been omitted.

## supplementary crystallographic information

### Comment

In the present work, the structure of *N*-(3-methylphenyl)-benzamide (N3MPBA) has been determined to explore the effect of substituents on the structures of benzanilides (Gowda *et al.*, 2003, 2008). The conformations of N—H and C=O bonds in the amide group of N3MPBA are *trans* to each other (Fig.1 ). Further, the conformation of the N—H bond is anti to the *meta*-methyl substituent in the aniline fragment, similar to that observed in *N*-(3-chlorophenyl)-benzamide (N3CPBA). The asymmetric unit of the structure contains four molecules. Aniline fragment in one of the molecules is disordered. The amide group –NHCO– forms dihedral angles of 24.4 (9)°, 22.3 (13)°, 22.7 (16)°, 25.2 (11)° with the benzoyl fragments, in the molecules 1, 2, 3, 4, respectively, compared to the value of 18.2 (2)° in N3CPBA (Gowda *et al.*, 2008). The dihedral angles between the two benzene rings (benzoyl and aniline fragments) in the four molecules are 70.8 (1)°, 70.6 (2)°, 73.3 (3)° and 74.2 (2)° for the molecules 1, 2, 3, 4, respectively, compared to the value of 61.0 (1)°.in N3CPBA. In the crystal structure, the molecules are linked into a chain through intermolecular N—H···O hydrogen bonds (Table 1 and Fig. 2).

### Experimental

The title compound was prepared according to the literature method (Gowda *et al.*, 2003). The purity of the compound was checked by determining its melting point. It was characterized by recording its infrared and NMR spectra. Single crystals of the title compound were obtained from an ethanolic solution and used for X-ray diffraction studies at room temperature.

### Refinement

In the absence of significant anomalous dispersion effects, Friedel pairs were merged and the Δf" terms were set to zero.

The amide H atoms were located in difference Fourier map but in the refinement process the N—H bond lengths wereas restrained to 0.86 (3) Å. For the other H atoms idealized geometry was assumed and they were refined using a riding model with C—H = 0.93–0.96 Å. All H atoms were refined with isotropic displacement parameters (*U*_iso_(NH) = 1.2, *U*_iso_(CH) = 1.2 and *U*_iso_(CH_3_) = 1.5 *U*_eq_ of the parent atom).

The benzene ring C29—C34 and the methyl group C42 are disordered over two nearly co-planar positions. The corresponding bond distances in the disordered groups were restrained to be equal. The site-occupancy factors were initially refined for the two positions of the methylphenyl fragment but they converged close to 0.5 and therefore the occupancy of the two positions was assumed to be equal 0.5. The U_ij_ values of the disordered atoms and those of C45 and C46 were restrained by ISOR instruction to approximate isotropic behaviour

### Crystal data

**Table:**

C_14_H_13_NO	*F*(000) = 1792
*M**_r_* = 211.25	*D*_x_ = 1.170 Mg m^−^^3^
Monoclinic, *C**c*	Cu *K*α radiation, λ = 1.54180 Å
Hall symbol: C -2yc	Cell parameters from 25 reflections
*a* = 13.269 (2) Å	θ = 3.3–20.6°
*b* = 53.686 (6) Å	µ = 0.58 mm^−^^1^
*c* = 9.3921 (12) Å	*T* = 299 K
β = 134.21 (1)°	Prism, colourless
*V* = 4795.7 (10) Å^3^	0.43 × 0.25 × 0.10 mm
*Z* = 16	

### Data collection

**Table:**

Enraf–Nonius CAD-4 diffractometer	*R*_int_ = 0.028
Radiation source: fine-focus sealed tube	θ_max_ = 67.0°, θ_min_ = 1.7°
graphite	*h* = −11→15
ω/2θ scans	*k* = −64→64
9315 measured reflections	*l* = −10→1
4168 independent reflections	3 standard reflections every 120 min
3197 reflections with *I* > 2σ(*I*)	intensity decay: 1.0%

### Refinement

**Table:**

Refinement on *F*^2^	Secondary atom site location: difference Fourier map
Least-squares matrix: full	Hydrogen site location: inferred from neighbouring sites
*R*[*F*^2^ > 2σ(*F*^2^)] = 0.044	H atoms treated by a mixture of independent and constrained refinement
*wR*(*F*^2^) = 0.129	*w* = 1/[σ^2^(*F*_o_^2^) + (0.0731*P*)^2^ + 0.8953*P*] where *P* = (*F*_o_^2^ + 2*F*_c_^2^)/3
*S* = 1.03	(Δ/σ)_max_ = 0.017
4168 reflections	Δρ_max_ = 0.27 e Å^−^^3^
653 parameters	Δρ_min_ = −0.13 e Å^−^^3^
110 restraints	Extinction correction: SHELXL97 (Sheldrick, 2008), Fc^*^=kFc[1+0.001xFc^2^λ^3^/sin(2θ)]^-1/4^
Primary atom site location: structure-invariant direct methods	Extinction coefficient: 0.00065 (10)

### Special details

**Table:**

Geometry. All e.s.d.'s (except the e.s.d. in the dihedral angle between two l.s. planes) are estimated using the full covariance matrix. The cell e.s.d.'s are taken into account individually in the estimation of e.s.d.'s in distances, angles and torsion angles; correlations between e.s.d.'s in cell parameters are only used when they are defined by crystal symmetry. An approximate (isotropic) treatment of cell e.s.d.'s is used for estimating e.s.d.'s involving l.s. planes.
Refinement. Refinement of *F*^2^ against ALL reflections. The weighted *R*-factor *wR* and goodness of fit *S* are based on *F*^2^, conventional *R*-factors *R* are based on *F*, with *F* set to zero for negative *F*^2^. The threshold expression of *F*^2^ > σ(*F*^2^) is used only for calculating *R*-factors(gt) *etc*. and is not relevant to the choice of reflections for refinement. *R*-factors based on *F*^2^ are statistically about twice as large as those based on *F*, and *R*- factors based on ALL data will be even larger.

### Fractional atomic coordinates and isotropic or equivalent isotropic displacement parameters (Å^2^)

**Table:**

	*x*	*y*	*z*	*U*_iso_*/*U*_eq_	Occ. (<1)
C1	0.0268 (4)	0.07396 (7)	0.1918 (5)	0.0628 (9)	
C2	0.1291 (5)	0.09159 (8)	0.2900 (7)	0.0769 (11)	
H2	0.1690	0.0962	0.2428	0.092*	
C3	0.1760 (5)	0.10310 (10)	0.4642 (8)	0.0960 (15)	
C4	0.1127 (6)	0.09577 (11)	0.5244 (8)	0.1005 (16)	
H4	0.1416	0.1030	0.6381	0.121*	
C5	0.0110 (7)	0.07874 (11)	0.4278 (8)	0.1040 (16)	
H5	−0.0301	0.0745	0.4738	0.125*	
C6	−0.0333 (5)	0.06749 (9)	0.2617 (6)	0.0814 (12)	
H6	−0.1037	0.0555	0.1956	0.098*	
C7	0.0673 (3)	0.05123 (7)	0.0101 (5)	0.0549 (8)	
C8	0.0021 (3)	0.03782 (6)	−0.1759 (5)	0.0526 (8)	
C9	0.0812 (4)	0.01993 (7)	−0.1675 (6)	0.0673 (10)	
H9	0.1729	0.0169	−0.0474	0.081*	
C10	0.0272 (4)	0.00661 (8)	−0.3325 (7)	0.0814 (12)	
H10	0.0814	−0.0057	−0.3225	0.098*	
C11	−0.1061 (4)	0.01127 (8)	−0.5119 (6)	0.0801 (12)	
H11	−0.1418	0.0025	−0.6243	0.096*	
C12	−0.1859 (4)	0.02881 (9)	−0.5248 (6)	0.0800 (12)	
H12	−0.2767	0.0319	−0.6465	0.096*	
C13	−0.1334 (4)	0.04215 (7)	−0.3580 (5)	0.0663 (10)	
H13	−0.1894	0.0540	−0.3686	0.080*	
C14	0.2931 (8)	0.12100 (15)	0.5724 (11)	0.155 (3)	
H14A	0.2686	0.1344	0.4853	0.232*	
H14B	0.3757	0.1127	0.6186	0.232*	
H14C	0.3114	0.1275	0.6838	0.232*	
O1	0.1948 (3)	0.05155 (6)	0.1495 (4)	0.0849 (9)	
N1	−0.0196 (3)	0.06211 (6)	0.0193 (4)	0.0606 (7)	
H1N	−0.109 (3)	0.0583 (7)	−0.064 (6)	0.073*	
C15	0.5271 (4)	0.07424 (7)	0.4130 (7)	0.0704 (10)	
C16	0.6297 (5)	0.09202 (8)	0.5177 (8)	0.0877 (13)	
H16	0.6687	0.0972	0.6419	0.105*	
C17	0.6765 (5)	0.10269 (9)	0.4285 (10)	0.0962 (16)	
C18	0.6156 (7)	0.09404 (11)	0.2474 (11)	0.1102 (17)	
H18	0.6482	0.1003	0.1933	0.132*	
C19	0.5128 (7)	0.07729 (12)	0.1419 (10)	0.1151 (18)	
H19	0.4723	0.0726	0.0159	0.138*	
C20	0.4667 (5)	0.06676 (10)	0.2261 (7)	0.0877 (13)	
H20	0.3959	0.0548	0.1563	0.105*	
C21	0.5657 (3)	0.05192 (7)	0.6757 (5)	0.0630 (9)	
C22	0.5001 (3)	0.03832 (7)	0.7316 (5)	0.0574 (8)	
C23	0.5806 (4)	0.02076 (8)	0.8826 (6)	0.0751 (11)	
H23	0.6730	0.0180	0.9454	0.090*	
C24	0.5267 (5)	0.00746 (9)	0.9406 (7)	0.0837 (12)	
H24	0.5818	−0.0044	1.0402	0.100*	
C25	0.3922 (5)	0.01154 (9)	0.8532 (7)	0.0870 (13)	
H25	0.3558	0.0026	0.8937	0.104*	
C26	0.3107 (4)	0.02906 (9)	0.7047 (7)	0.0843 (13)	
H26	0.2194	0.0320	0.6460	0.101*	
C27	0.3634 (4)	0.04224 (8)	0.6425 (6)	0.0695 (10)	
H27	0.3069	0.0538	0.5404	0.083*	
C28	0.7952 (8)	0.12088 (14)	0.5522 (13)	0.151 (3)	
H28A	0.7679	0.1361	0.4794	0.227*	
H28B	0.8758	0.1140	0.5843	0.227*	
H28C	0.8181	0.1242	0.6725	0.227*	
O2	0.6934 (2)	0.05281 (7)	0.7906 (4)	0.0939 (10)	
N2	0.4806 (3)	0.06267 (6)	0.4954 (5)	0.0649 (8)	
H2N	0.395 (3)	0.0596 (7)	0.418 (6)	0.078*	
C29A	0.8798 (11)	0.1769 (2)	0.9070 (18)	0.056 (3)	0.50
C30A	0.7568 (10)	0.18485 (19)	0.7249 (15)	0.070 (3)	0.50
H30A	0.7566	0.1976	0.6577	0.084*	0.50
C31A	0.6348 (10)	0.1735 (2)	0.6450 (17)	0.084 (3)	0.50
H31A	0.5507	0.1784	0.5202	0.101*	0.50
C32A	0.6326 (13)	0.1549 (3)	0.744 (2)	0.091 (4)	0.50
H32A	0.5472	0.1477	0.6853	0.110*	0.50
C33A	0.7536 (17)	0.1469 (3)	0.928 (3)	0.090 (4)	0.50
C34A	0.8778 (10)	0.15780 (17)	1.0046 (14)	0.066 (2)	0.50
H34A	0.9625	0.1522	1.1255	0.079*	0.50
C29B	0.9176 (10)	0.1711 (2)	0.9772 (16)	0.061 (3)	0.50
C30B	0.9455 (11)	0.15245 (18)	1.0998 (16)	0.076 (2)	0.50
H30B	1.0376	0.1490	1.2176	0.091*	0.50
C31B	0.8368 (13)	0.1389 (2)	1.047 (2)	0.103 (3)	0.50
H31B	0.8527	0.1265	1.1305	0.124*	0.50
C32B	0.7039 (18)	0.1440 (3)	0.869 (3)	0.100 (5)	0.50
H32B	0.6312	0.1341	0.8321	0.120*	0.50
C33B	0.6700 (13)	0.1622 (3)	0.744 (2)	0.084 (3)	0.50
C34B	0.7839 (13)	0.1759 (2)	0.8012 (19)	0.072 (3)	0.50
H34B	0.7679	0.1884	0.7185	0.087*	0.50
C35	1.1118 (4)	0.19803 (7)	1.1899 (5)	0.0628 (9)	
C36	1.2296 (4)	0.21224 (6)	1.2413 (5)	0.0563 (8)	
C37	1.2951 (4)	0.22996 (8)	1.3885 (6)	0.0747 (11)	
H37	1.2638	0.2328	1.4501	0.090*	
C38	1.4067 (5)	0.24351 (9)	1.4454 (7)	0.0876 (13)	
H38	1.4507	0.2554	1.5454	0.105*	
C39	1.4528 (5)	0.23942 (9)	1.3541 (7)	0.0830 (12)	
H39	1.5278	0.2485	1.3918	0.100*	
C40	1.3880 (5)	0.22196 (9)	1.2081 (7)	0.0805 (12)	
H40	1.4204	0.2190	1.1482	0.097*	
C41	1.2753 (4)	0.20854 (7)	1.1477 (6)	0.0664 (10)	
H41	1.2300	0.1971	1.0448	0.080*	
C42A	0.7536 (14)	0.1263 (2)	1.037 (2)	0.128 (4)	0.50
H42A	0.7881	0.1327	1.1597	0.153*	0.50
H42B	0.8131	0.1130	1.0635	0.153*	0.50
H42C	0.6596	0.1202	0.9565	0.153*	0.50
C42B	0.5236 (13)	0.1672 (2)	0.5592 (18)	0.124 (4)	0.50
H42D	0.4725	0.1729	0.5907	0.149*	0.50
H42E	0.4812	0.1522	0.4817	0.149*	0.50
H42F	0.5214	0.1798	0.4845	0.149*	0.50
O3	1.0999 (4)	0.19713 (7)	1.3092 (5)	0.0986 (11)	
N3	1.0189 (4)	0.18720 (6)	1.0112 (5)	0.0688 (8)	
H3N	1.008 (5)	0.1884 (8)	0.910 (5)	0.083*	
C43	0.1504 (4)	0.17535 (7)	0.6956 (6)	0.0675 (10)	
C44	0.1398 (6)	0.15736 (8)	0.7880 (7)	0.0833 (12)	
H44	0.0521	0.1527	0.7365	0.100*	
C45	0.2631 (7)	0.14582 (10)	0.9623 (8)	0.1007 (17)	
C46	0.3895 (7)	0.15302 (12)	1.0333 (9)	0.1094 (18)	
H46	0.4709	0.1457	1.1493	0.131*	
C47	0.4011 (6)	0.17042 (12)	0.9410 (9)	0.1085 (18)	
H47	0.4887	0.1747	0.9905	0.130*	
C48	0.2805 (5)	0.18180 (10)	0.7720 (7)	0.0887 (13)	
H48	0.2876	0.1940	0.7091	0.106*	
C49	−0.0664 (4)	0.19878 (7)	0.5125 (5)	0.0623 (9)	
C50	−0.1861 (4)	0.21174 (7)	0.3245 (5)	0.0564 (8)	
C51	−0.2317 (4)	0.20666 (8)	0.1450 (6)	0.0739 (11)	
H51	−0.1860	0.1946	0.1358	0.089*	
C52	−0.3455 (5)	0.21933 (10)	−0.0237 (6)	0.0897 (14)	
H52	−0.3767	0.2155	−0.1457	0.108*	
C53	−0.4119 (5)	0.23724 (9)	−0.0131 (7)	0.0860 (13)	
H53	−0.4882	0.2457	−0.1270	0.103*	
C54	−0.3655 (5)	0.24283 (9)	0.1677 (7)	0.0828 (12)	
H54	−0.4092	0.2553	0.1770	0.099*	
C55	−0.2547 (4)	0.22996 (8)	0.3339 (6)	0.0706 (10)	
H55	−0.2252	0.2335	0.4551	0.085*	
C56	0.2485 (9)	0.12685 (13)	1.0643 (11)	0.147 (3)	
H56A	0.1941	0.1130	0.9767	0.221*	
H56B	0.2021	0.1343	1.0988	0.221*	
H56C	0.3401	0.1211	1.1826	0.221*	
O4	−0.0545 (3)	0.19914 (7)	0.6551 (4)	0.0918 (9)	
N4	0.0282 (3)	0.18719 (6)	0.5235 (5)	0.0661 (8)	
H4N	0.026 (5)	0.1923 (8)	0.439 (6)	0.079*	

### Atomic displacement parameters (Å^2^)

**Table:**

	*U*^11^	*U*^22^	*U*^33^	*U*^12^	*U*^13^	*U*^23^
C1	0.0554 (19)	0.060 (2)	0.0539 (19)	0.0061 (16)	0.0311 (17)	−0.0023 (16)
C2	0.076 (3)	0.071 (2)	0.078 (3)	−0.005 (2)	0.052 (2)	−0.010 (2)
C3	0.087 (3)	0.079 (3)	0.089 (3)	−0.010 (2)	0.050 (3)	−0.020 (2)
C4	0.116 (4)	0.110 (4)	0.084 (3)	−0.006 (3)	0.073 (3)	−0.017 (3)
C5	0.115 (4)	0.122 (4)	0.091 (3)	−0.004 (4)	0.078 (3)	−0.017 (3)
C6	0.086 (3)	0.099 (3)	0.073 (3)	−0.007 (2)	0.061 (2)	−0.013 (2)
C7	0.0422 (17)	0.066 (2)	0.0504 (18)	−0.0015 (14)	0.0302 (16)	0.0017 (15)
C8	0.0417 (15)	0.0613 (18)	0.0519 (18)	−0.0001 (14)	0.0316 (14)	0.0067 (15)
C9	0.0509 (18)	0.079 (2)	0.063 (2)	0.0065 (17)	0.0369 (18)	−0.0004 (19)
C10	0.067 (2)	0.088 (3)	0.090 (3)	0.002 (2)	0.055 (2)	−0.016 (2)
C11	0.071 (2)	0.093 (3)	0.071 (3)	−0.013 (2)	0.048 (2)	−0.026 (2)
C12	0.059 (2)	0.098 (3)	0.055 (2)	0.001 (2)	0.0288 (18)	−0.012 (2)
C13	0.0500 (18)	0.074 (2)	0.058 (2)	0.0077 (16)	0.0314 (17)	−0.0004 (18)
C14	0.156 (6)	0.148 (6)	0.150 (7)	−0.074 (5)	0.103 (6)	−0.075 (5)
O1	0.0409 (13)	0.134 (3)	0.0570 (15)	0.0001 (14)	0.0259 (12)	−0.0183 (16)
N1	0.0432 (13)	0.0758 (19)	0.0517 (15)	−0.0011 (13)	0.0290 (12)	−0.0077 (14)
C15	0.0543 (19)	0.066 (2)	0.084 (3)	0.0085 (17)	0.046 (2)	0.011 (2)
C16	0.066 (2)	0.074 (3)	0.113 (4)	0.005 (2)	0.058 (3)	0.009 (3)
C17	0.083 (3)	0.069 (3)	0.140 (5)	0.000 (2)	0.079 (3)	0.002 (3)
C18	0.122 (5)	0.105 (4)	0.125 (5)	−0.002 (4)	0.094 (4)	0.004 (4)
C19	0.126 (5)	0.110 (4)	0.120 (5)	−0.004 (4)	0.090 (4)	−0.003 (4)
C20	0.087 (3)	0.099 (3)	0.087 (3)	0.000 (2)	0.064 (3)	0.008 (3)
C21	0.0408 (18)	0.076 (2)	0.0552 (19)	−0.0005 (16)	0.0271 (16)	−0.0074 (18)
C22	0.0439 (15)	0.070 (2)	0.0473 (17)	0.0005 (15)	0.0278 (15)	−0.0066 (16)
C23	0.0524 (19)	0.089 (3)	0.063 (2)	0.0095 (19)	0.0322 (18)	0.004 (2)
C24	0.069 (2)	0.092 (3)	0.068 (2)	0.002 (2)	0.040 (2)	0.014 (2)
C25	0.073 (2)	0.094 (3)	0.078 (3)	−0.009 (2)	0.046 (2)	0.004 (2)
C26	0.058 (2)	0.109 (3)	0.088 (3)	0.003 (2)	0.051 (2)	0.015 (3)
C27	0.0481 (18)	0.080 (2)	0.065 (2)	0.0057 (17)	0.0341 (18)	0.0065 (19)
C28	0.150 (6)	0.127 (5)	0.203 (8)	−0.053 (5)	0.132 (6)	−0.033 (5)
O2	0.0388 (14)	0.156 (3)	0.0672 (17)	−0.0020 (15)	0.0299 (13)	0.0121 (18)
N2	0.0403 (13)	0.081 (2)	0.0593 (18)	−0.0014 (14)	0.0295 (14)	0.0028 (15)
C29A	0.064 (6)	0.057 (5)	0.061 (6)	0.002 (5)	0.048 (5)	0.006 (5)
C30A	0.066 (5)	0.067 (5)	0.067 (5)	−0.002 (4)	0.042 (4)	0.011 (4)
C31A	0.060 (5)	0.096 (6)	0.084 (5)	−0.006 (4)	0.045 (4)	0.017 (5)
C32A	0.078 (6)	0.088 (7)	0.102 (7)	−0.014 (5)	0.060 (6)	0.018 (6)
C33A	0.089 (8)	0.090 (7)	0.091 (8)	−0.002 (6)	0.062 (6)	0.016 (6)
C34A	0.067 (5)	0.059 (4)	0.062 (5)	−0.001 (4)	0.041 (4)	0.012 (4)
C29B	0.060 (5)	0.065 (6)	0.062 (6)	0.002 (5)	0.044 (5)	−0.007 (5)
C30B	0.076 (5)	0.078 (5)	0.079 (6)	0.006 (4)	0.056 (5)	0.014 (5)
C31B	0.107 (7)	0.095 (6)	0.107 (7)	−0.011 (5)	0.075 (6)	0.002 (5)
C32B	0.094 (8)	0.092 (8)	0.107 (9)	−0.015 (6)	0.067 (7)	−0.005 (7)
C33B	0.080 (6)	0.077 (7)	0.087 (6)	−0.009 (5)	0.056 (5)	0.005 (5)
C34B	0.077 (6)	0.071 (6)	0.069 (6)	−0.003 (5)	0.051 (5)	0.001 (5)
C35	0.069 (2)	0.073 (2)	0.059 (2)	0.0066 (18)	0.049 (2)	0.0058 (18)
C36	0.0578 (18)	0.066 (2)	0.0483 (17)	0.0068 (16)	0.0382 (16)	0.0051 (16)
C37	0.087 (3)	0.089 (3)	0.063 (2)	−0.004 (2)	0.057 (2)	−0.013 (2)
C38	0.098 (3)	0.096 (3)	0.071 (3)	−0.027 (3)	0.060 (3)	−0.024 (2)
C39	0.084 (3)	0.092 (3)	0.074 (3)	−0.027 (2)	0.056 (2)	−0.009 (2)
C40	0.087 (3)	0.095 (3)	0.086 (3)	−0.015 (2)	0.070 (3)	−0.009 (2)
C41	0.076 (2)	0.071 (2)	0.069 (2)	−0.0065 (18)	0.056 (2)	−0.0071 (18)
C42A	0.121 (7)	0.127 (8)	0.148 (8)	−0.017 (6)	0.099 (6)	0.040 (6)
C42B	0.103 (7)	0.129 (7)	0.105 (7)	−0.012 (6)	0.059 (5)	0.007 (6)
O3	0.102 (2)	0.154 (3)	0.0793 (18)	−0.024 (2)	0.0777 (19)	−0.0162 (19)
N3	0.0747 (19)	0.082 (2)	0.069 (2)	−0.0147 (16)	0.0574 (18)	−0.0115 (17)
C43	0.080 (3)	0.070 (2)	0.058 (2)	0.0092 (19)	0.050 (2)	−0.0008 (18)
C44	0.106 (3)	0.071 (3)	0.075 (3)	0.013 (2)	0.064 (3)	0.001 (2)
C45	0.142 (5)	0.078 (3)	0.085 (3)	0.027 (3)	0.081 (3)	0.015 (2)
C46	0.100 (4)	0.118 (4)	0.090 (4)	0.035 (3)	0.059 (3)	0.009 (3)
C47	0.090 (3)	0.129 (5)	0.086 (3)	0.029 (3)	0.054 (3)	0.007 (3)
C48	0.080 (3)	0.102 (3)	0.083 (3)	0.018 (2)	0.056 (3)	0.003 (3)
C49	0.067 (2)	0.076 (2)	0.056 (2)	−0.0033 (18)	0.0470 (19)	−0.0041 (17)
C50	0.0577 (18)	0.066 (2)	0.0540 (19)	−0.0034 (15)	0.0419 (17)	−0.0022 (16)
C51	0.085 (3)	0.085 (3)	0.061 (2)	0.012 (2)	0.054 (2)	0.001 (2)
C52	0.094 (3)	0.117 (4)	0.059 (2)	0.021 (3)	0.054 (2)	0.008 (2)
C53	0.083 (3)	0.109 (3)	0.070 (3)	0.022 (2)	0.055 (2)	0.028 (2)
C54	0.085 (3)	0.091 (3)	0.091 (3)	0.017 (2)	0.068 (3)	0.012 (3)
C55	0.072 (2)	0.085 (3)	0.064 (2)	0.005 (2)	0.051 (2)	0.000 (2)
C56	0.187 (7)	0.116 (5)	0.132 (6)	0.036 (5)	0.109 (6)	0.049 (4)
O4	0.090 (2)	0.141 (3)	0.0584 (16)	0.0276 (19)	0.0568 (16)	0.0143 (17)
N4	0.0693 (18)	0.081 (2)	0.0593 (18)	0.0106 (16)	0.0490 (16)	0.0079 (15)

### Geometric parameters (Å, °)

**Table:**

C1—C2	1.357 (6)	C32A—H32A	0.9300
C1—C6	1.385 (6)	C33A—C34A	1.386 (17)
C1—N1	1.420 (5)	C33A—C42A	1.503 (16)
C2—C3	1.423 (7)	C34A—H34A	0.9300
C2—H2	0.9300	C29B—C34B	1.362 (13)
C3—C4	1.359 (8)	C29B—C30B	1.367 (13)
C3—C14	1.472 (8)	C29B—N3	1.435 (11)
C4—C5	1.331 (8)	C30B—C31B	1.365 (13)
C4—H4	0.9300	C30B—H30B	0.9300
C5—C6	1.366 (7)	C31B—C32B	1.367 (16)
C5—H5	0.9300	C31B—H31B	0.9300
C6—H6	0.9300	C32B—C33B	1.344 (16)
C7—O1	1.219 (4)	C32B—H32B	0.9300
C7—N1	1.348 (4)	C33B—C34B	1.407 (16)
C7—C8	1.487 (5)	C33B—C42B	1.468 (16)
C8—C9	1.384 (5)	C34B—H34B	0.9300
C8—C13	1.387 (5)	C35—O3	1.236 (5)
C9—C10	1.371 (6)	C35—N3	1.338 (5)
C9—H9	0.9300	C35—C36	1.486 (5)
C10—C11	1.369 (6)	C36—C37	1.377 (5)
C10—H10	0.9300	C36—C41	1.386 (5)
C11—C12	1.358 (6)	C37—C38	1.380 (6)
C11—H11	0.9300	C37—H37	0.9300
C12—C13	1.389 (6)	C38—C39	1.374 (7)
C12—H12	0.9300	C38—H38	0.9300
C13—H13	0.9300	C39—C40	1.362 (6)
C14—H14A	0.9600	C39—H39	0.9300
C14—H14B	0.9600	C40—C41	1.376 (6)
C14—H14C	0.9600	C40—H40	0.9300
N1—H1N	0.87 (3)	C41—H41	0.9300
C15—C16	1.366 (6)	C42A—H42A	0.9600
C15—C20	1.387 (6)	C42A—H42B	0.9600
C15—N2	1.426 (5)	C42A—H42C	0.9600
C16—C17	1.463 (8)	C42B—H42D	0.9600
C16—H16	0.9300	C42B—H42E	0.9600
C17—C18	1.359 (8)	C42B—H42F	0.9600
C17—C28	1.494 (8)	N3—H3N	0.86 (3)
C18—C19	1.329 (8)	C43—C44	1.370 (6)
C18—H18	0.9300	C43—C48	1.373 (7)
C19—C20	1.413 (8)	C43—N4	1.411 (5)
C19—H19	0.9300	C44—C45	1.416 (7)
C20—H20	0.9300	C44—H44	0.9300
C21—O2	1.219 (4)	C45—C46	1.360 (9)
C21—N2	1.345 (5)	C45—C56	1.500 (9)
C21—C22	1.484 (5)	C46—C47	1.353 (9)
C22—C27	1.385 (5)	C46—H46	0.9300
C22—C23	1.389 (5)	C47—C48	1.382 (7)
C23—C24	1.364 (6)	C47—H47	0.9300
C23—H23	0.9300	C48—H48	0.9300
C24—C25	1.365 (6)	C49—O4	1.234 (4)
C24—H24	0.9300	C49—N4	1.339 (5)
C25—C26	1.377 (6)	C49—C50	1.488 (5)
C25—H25	0.9300	C50—C51	1.365 (5)
C26—C27	1.378 (6)	C50—C55	1.380 (5)
C26—H26	0.9300	C51—C52	1.384 (6)
C27—H27	0.9300	C51—H51	0.9300
C28—H28A	0.9600	C52—C53	1.354 (7)
C28—H28B	0.9600	C52—H52	0.9300
C28—H28C	0.9600	C53—C54	1.376 (7)
N2—H2N	0.84 (3)	C53—H53	0.9300
C29A—C30A	1.371 (13)	C54—C55	1.369 (6)
C29A—C34A	1.386 (12)	C54—H54	0.9300
C29A—N3	1.467 (10)	C55—H55	0.9300
C30A—C31A	1.365 (12)	C56—H56A	0.9600
C30A—H30A	0.9300	C56—H56B	0.9600
C31A—C32A	1.379 (14)	C56—H56C	0.9600
C31A—H31A	0.9300	N4—H4N	0.82 (3)
C32A—C33A	1.371 (15)		
			
C2—C1—C6	119.0 (4)	C32A—C33A—C42A	122.1 (13)
C2—C1—N1	121.4 (4)	C34A—C33A—C42A	121.4 (13)
C6—C1—N1	119.6 (4)	C33A—C34A—C29A	122.2 (10)
C1—C2—C3	120.9 (5)	C33A—C34A—H34A	118.9
C1—C2—H2	119.6	C29A—C34A—H34A	118.9
C3—C2—H2	119.6	C34B—C29B—C30B	121.3 (10)
C4—C3—C2	116.8 (5)	C34B—C29B—N3	112.4 (9)
C4—C3—C14	124.3 (6)	C30B—C29B—N3	126.3 (8)
C2—C3—C14	118.8 (6)	C31B—C30B—C29B	119.3 (11)
C5—C4—C3	122.9 (5)	C31B—C30B—H30B	120.3
C5—C4—H4	118.6	C29B—C30B—H30B	120.3
C3—C4—H4	118.6	C30B—C31B—C32B	118.0 (13)
C4—C5—C6	120.4 (6)	C30B—C31B—H31B	121.0
C4—C5—H5	119.8	C32B—C31B—H31B	121.0
C6—C5—H5	119.8	C33B—C32B—C31B	125.2 (16)
C5—C6—C1	120.0 (5)	C33B—C32B—H32B	117.4
C5—C6—H6	120.0	C31B—C32B—H32B	117.4
C1—C6—H6	120.0	C32B—C33B—C34B	115.5 (13)
O1—C7—N1	122.2 (3)	C32B—C33B—C42B	121.8 (14)
O1—C7—C8	120.3 (3)	C34B—C33B—C42B	122.6 (13)
N1—C7—C8	117.5 (3)	C29B—C34B—C33B	120.5 (11)
C9—C8—C13	117.8 (3)	C29B—C34B—H34B	119.8
C9—C8—C7	118.2 (3)	C33B—C34B—H34B	119.8
C13—C8—C7	124.0 (3)	O3—C35—N3	122.1 (4)
C10—C9—C8	121.4 (3)	O3—C35—C36	119.8 (4)
C10—C9—H9	119.3	N3—C35—C36	118.1 (3)
C8—C9—H9	119.3	C37—C36—C41	119.2 (4)
C11—C10—C9	120.3 (4)	C37—C36—C35	117.7 (3)
C11—C10—H10	119.9	C41—C36—C35	123.1 (3)
C9—C10—H10	119.9	C36—C37—C38	120.5 (4)
C12—C11—C10	119.7 (4)	C36—C37—H37	119.7
C12—C11—H11	120.2	C38—C37—H37	119.7
C10—C11—H11	120.2	C39—C38—C37	119.9 (4)
C11—C12—C13	120.7 (4)	C39—C38—H38	120.0
C11—C12—H12	119.7	C37—C38—H38	120.0
C13—C12—H12	119.7	C40—C39—C38	119.7 (4)
C8—C13—C12	120.2 (4)	C40—C39—H39	120.2
C8—C13—H13	119.9	C38—C39—H39	120.2
C12—C13—H13	119.9	C39—C40—C41	121.1 (4)
C3—C14—H14A	109.5	C39—C40—H40	119.4
C3—C14—H14B	109.5	C41—C40—H40	119.4
H14A—C14—H14B	109.5	C40—C41—C36	119.6 (4)
C3—C14—H14C	109.5	C40—C41—H41	120.2
H14A—C14—H14C	109.5	C36—C41—H41	120.2
H14B—C14—H14C	109.5	C33A—C42A—H42A	109.5
C7—N1—C1	123.9 (3)	C33A—C42A—H42B	109.5
C7—N1—H1N	121 (3)	H42A—C42A—H42B	109.5
C1—N1—H1N	112 (3)	C33A—C42A—H42C	109.5
C16—C15—C20	121.3 (5)	H42A—C42A—H42C	109.5
C16—C15—N2	120.3 (4)	H42B—C42A—H42C	109.5
C20—C15—N2	118.3 (4)	C33B—C42B—H42D	109.5
C15—C16—C17	118.0 (5)	C33B—C42B—H42E	109.5
C15—C16—H16	121.0	H42D—C42B—H42E	109.5
C17—C16—H16	121.0	C33B—C42B—H42F	109.5
C18—C17—C16	117.6 (5)	H42D—C42B—H42F	109.5
C18—C17—C28	125.5 (6)	H42E—C42B—H42F	109.5
C16—C17—C28	116.7 (6)	C35—N3—C29B	116.2 (6)
C19—C18—C17	124.7 (6)	C35—N3—C29A	129.2 (6)
C19—C18—H18	117.6	C35—N3—H3N	131 (3)
C17—C18—H18	117.6	C29B—N3—H3N	113 (3)
C18—C19—C20	118.3 (6)	C29A—N3—H3N	97 (3)
C18—C19—H19	120.9	C44—C43—C48	119.6 (4)
C20—C19—H19	120.9	C44—C43—N4	120.0 (4)
C15—C20—C19	120.0 (5)	C48—C43—N4	120.4 (4)
C15—C20—H20	120.0	C43—C44—C45	119.6 (5)
C19—C20—H20	120.0	C43—C44—H44	120.2
O2—C21—N2	121.7 (4)	C45—C44—H44	120.2
O2—C21—C22	120.1 (3)	C46—C45—C44	118.6 (5)
N2—C21—C22	118.1 (3)	C46—C45—C56	123.0 (6)
C27—C22—C23	118.1 (4)	C44—C45—C56	118.4 (6)
C27—C22—C21	123.8 (3)	C47—C46—C45	122.3 (6)
C23—C22—C21	118.2 (3)	C47—C46—H46	118.9
C24—C23—C22	121.3 (4)	C45—C46—H46	118.9
C24—C23—H23	119.4	C46—C47—C48	118.9 (6)
C22—C23—H23	119.4	C46—C47—H47	120.6
C23—C24—C25	120.4 (4)	C48—C47—H47	120.6
C23—C24—H24	119.8	C43—C48—C47	121.0 (6)
C25—C24—H24	119.8	C43—C48—H48	119.5
C24—C25—C26	119.5 (5)	C47—C48—H48	119.5
C24—C25—H25	120.3	O4—C49—N4	121.3 (3)
C26—C25—H25	120.3	O4—C49—C50	120.2 (3)
C25—C26—C27	120.5 (4)	N4—C49—C50	118.5 (3)
C25—C26—H26	119.7	C51—C50—C55	118.5 (4)
C27—C26—H26	119.7	C51—C50—C49	124.0 (3)
C26—C27—C22	120.2 (4)	C55—C50—C49	117.5 (3)
C26—C27—H27	119.9	C50—C51—C52	120.4 (4)
C22—C27—H27	119.9	C50—C51—H51	119.8
C17—C28—H28A	109.5	C52—C51—H51	119.8
C17—C28—H28B	109.5	C53—C52—C51	120.7 (4)
H28A—C28—H28B	109.5	C53—C52—H52	119.6
C17—C28—H28C	109.5	C51—C52—H52	119.6
H28A—C28—H28C	109.5	C52—C53—C54	119.4 (4)
H28B—C28—H28C	109.5	C52—C53—H53	120.3
C21—N2—C15	124.7 (3)	C54—C53—H53	120.3
C21—N2—H2N	116 (3)	C55—C54—C53	119.8 (4)
C15—N2—H2N	117 (3)	C55—C54—H54	120.1
C30A—C29A—C34A	120.1 (9)	C53—C54—H54	120.1
C30A—C29A—N3	124.2 (8)	C54—C55—C50	121.1 (4)
C34A—C29A—N3	115.7 (9)	C54—C55—H55	119.5
C31A—C30A—C29A	118.0 (10)	C50—C55—H55	119.5
C31A—C30A—H30A	121.0	C45—C56—H56A	109.5
C29A—C30A—H30A	121.0	C45—C56—H56B	109.5
C30A—C31A—C32A	121.9 (11)	H56A—C56—H56B	109.5
C30A—C31A—H31A	119.1	C45—C56—H56C	109.5
C32A—C31A—H31A	119.1	H56A—C56—H56C	109.5
C33A—C32A—C31A	121.3 (12)	H56B—C56—H56C	109.5
C33A—C32A—H32A	119.3	C49—N4—C43	124.4 (3)
C31A—C32A—H32A	119.3	C49—N4—H4N	112 (3)
C32A—C33A—C34A	116.5 (11)	C43—N4—H4N	120 (3)
			
C6—C1—C2—C3	0.9 (6)	N3—C29A—C34A—C33A	177.5 (13)
N1—C1—C2—C3	−179.1 (4)	C34B—C29B—C30B—C31B	−2.0 (19)
C1—C2—C3—C4	−0.7 (7)	N3—C29B—C30B—C31B	179.2 (11)
C1—C2—C3—C14	176.3 (6)	C29B—C30B—C31B—C32B	2(2)
C2—C3—C4—C5	−0.2 (8)	C30B—C31B—C32B—C33B	−3(3)
C14—C3—C4—C5	−177.0 (7)	C31B—C32B—C33B—C34B	3(3)
C3—C4—C5—C6	0.8 (9)	C31B—C32B—C33B—C42B	−177.0 (17)
C4—C5—C6—C1	−0.6 (8)	C30B—C29B—C34B—C33B	2(2)
C2—C1—C6—C5	−0.3 (7)	N3—C29B—C34B—C33B	−179.1 (10)
N1—C1—C6—C5	179.8 (4)	C32B—C33B—C34B—C29B	−2(2)
O1—C7—C8—C9	19.4 (5)	C42B—C33B—C34B—C29B	177.6 (13)
N1—C7—C8—C9	−158.6 (3)	O3—C35—C36—C37	−19.7 (5)
O1—C7—C8—C13	−160.5 (4)	N3—C35—C36—C37	158.2 (4)
N1—C7—C8—C13	21.6 (5)	O3—C35—C36—C41	160.7 (4)
C13—C8—C9—C10	−1.0 (6)	N3—C35—C36—C41	−21.5 (5)
C7—C8—C9—C10	179.1 (4)	C41—C36—C37—C38	−1.4 (6)
C8—C9—C10—C11	1.8 (7)	C35—C36—C37—C38	178.9 (4)
C9—C10—C11—C12	−1.5 (7)	C36—C37—C38—C39	0.3 (7)
C10—C11—C12—C13	0.3 (7)	C37—C38—C39—C40	−0.1 (7)
C9—C8—C13—C12	−0.1 (6)	C38—C39—C40—C41	1.1 (7)
C7—C8—C13—C12	179.7 (4)	C39—C40—C41—C36	−2.1 (7)
C11—C12—C13—C8	0.5 (7)	C37—C36—C41—C40	2.3 (6)
O1—C7—N1—C1	−2.7 (6)	C35—C36—C41—C40	−178.1 (4)
C8—C7—N1—C1	175.2 (3)	O3—C35—N3—C29B	−9.9 (8)
C2—C1—N1—C7	51.7 (5)	C36—C35—N3—C29B	172.3 (6)
C6—C1—N1—C7	−128.3 (4)	O3—C35—N3—C29A	12.1 (9)
C20—C15—C16—C17	−1.2 (6)	C36—C35—N3—C29A	−165.7 (6)
N2—C15—C16—C17	178.1 (4)	C34B—C29B—N3—C35	131.8 (9)
C15—C16—C17—C18	−0.6 (7)	C30B—C29B—N3—C35	−49.3 (14)
C15—C16—C17—C28	−176.2 (5)	C34B—C29B—N3—C29A	1.2 (18)
C16—C17—C18—C19	2.7 (9)	C30A—C29A—N3—C35	120.8 (12)
C28—C17—C18—C19	177.9 (7)	C34A—C29A—N3—C35	−59.3 (13)
C17—C18—C19—C20	−2.9 (10)	C30A—C29A—N3—C29B	−178 (4)
C16—C15—C20—C19	1.0 (7)	C34A—C29A—N3—C29B	2.2 (18)
N2—C15—C20—C19	−178.2 (4)	C48—C43—C44—C45	0.7 (6)
C18—C19—C20—C15	0.9 (8)	N4—C43—C44—C45	−179.1 (4)
O2—C21—C22—C27	160.0 (4)	C43—C44—C45—C46	0.0 (7)
N2—C21—C22—C27	−21.1 (5)	C43—C44—C45—C56	177.8 (5)
O2—C21—C22—C23	−19.6 (5)	C44—C45—C46—C47	−1.3 (9)
N2—C21—C22—C23	159.3 (4)	C56—C45—C46—C47	−179.0 (6)
C27—C22—C23—C24	0.8 (6)	C45—C46—C47—C48	2.0 (9)
C21—C22—C23—C24	−179.6 (4)	C44—C43—C48—C47	0.0 (7)
C22—C23—C24—C25	−1.3 (7)	N4—C43—C48—C47	179.7 (4)
C23—C24—C25—C26	0.5 (7)	C46—C47—C48—C43	−1.2 (8)
C24—C25—C26—C27	0.8 (8)	O4—C49—C50—C51	−160.1 (4)
C25—C26—C27—C22	−1.2 (7)	N4—C49—C50—C51	21.0 (6)
C23—C22—C27—C26	0.5 (6)	O4—C49—C50—C55	19.7 (5)
C21—C22—C27—C26	−179.1 (4)	N4—C49—C50—C55	−159.2 (4)
O2—C21—N2—C15	5.2 (6)	C55—C50—C51—C52	−0.9 (6)
C22—C21—N2—C15	−173.6 (3)	C49—C50—C51—C52	179.0 (4)
C16—C15—N2—C21	−52.2 (5)	C50—C51—C52—C53	1.2 (8)
C20—C15—N2—C21	127.1 (4)	C51—C52—C53—C54	−0.1 (8)
C34A—C29A—C30A—C31A	0.0 (19)	C52—C53—C54—C55	−1.2 (7)
N3—C29A—C30A—C31A	179.9 (10)	C53—C54—C55—C50	1.5 (7)
C29A—C30A—C31A—C32A	1.5 (19)	C51—C50—C55—C54	−0.4 (6)
C30A—C31A—C32A—C33A	0(2)	C49—C50—C55—C54	179.7 (4)
C31A—C32A—C33A—C34A	−2(3)	O4—C49—N4—C43	−2.4 (6)
C31A—C32A—C33A—C42A	−179.0 (16)	C50—C49—N4—C43	176.5 (4)
C32A—C33A—C34A—C29A	3(3)	C44—C43—N4—C49	54.9 (5)
C42A—C33A—C34A—C29A	−179.5 (15)	C48—C43—N4—C49	−124.8 (5)
C30A—C29A—C34A—C33A	−3(2)		

### Hydrogen-bond geometry (Å, °)

**Table:**

*D*—H···*A*	*D*—H	H···*A*	*D*···*A*	*D*—H···*A*
N1—H1N···O2^i^	0.87 (3)	1.96 (3)	2.822 (4)	168 (4)
N2—H2N···O1	0.84 (3)	2.06 (3)	2.848 (4)	156 (5)
N3—H3N···O4^ii^	0.86 (3)	2.00 (3)	2.829 (4)	163 (4)
N4—H4N···O3^i^	0.82 (3)	2.04 (3)	2.813 (4)	156 (5)

Symmetry codes: (i) *x*−1, *y*, *z*−1; (ii) *x*+1, *y*, *z*.
